# p53: From Fundamental Biology to Clinical Applications in Cancer

**DOI:** 10.3390/biology11091325

**Published:** 2022-09-06

**Authors:** Maurizio Capuozzo, Mariachiara Santorsola, Marco Bocchetti, Francesco Perri, Marco Cascella, Vincenza Granata, Venere Celotto, Oreste Gualillo, Alessia Maria Cossu, Guglielmo Nasti, Michele Caraglia, Alessandro Ottaiano

**Affiliations:** 1 Coordinamento Farmaceutico, ASL-Naples-3, 80056 Ercolano, Italy; 2 Istituto Nazionale Tumori di Napoli, IRCCS “G. Pascale”, Via M. Semmola, 80131 Naples, Italy; 3 Department of Precision Medicine, University of Campania “L. Vanvitelli”, Via L. De Crecchio 7, 80138 Naples, Italy; 4 Laboratory of Molecular and Precision Oncology, Biogem Scarl, Institute of Genetic Research, 83031 Ariano Irpino, Italy; 5 SERGAS (Servizo Galego de Saude) and IDIS (Instituto de Investigación Sanitaria de Santiago), NEIRID Lab (Neuroendocrine Interactions in Rheumatology and Inflammatory Diseases), Research Laboratory 9, Santiago University Clinical Hospital, 15706 Santiago de Compostela, Spain

**Keywords:** p53, tp53, cancer, DNA repair, tumour progression

## Abstract

**Simple Summary:**

p53 tumour suppressor gene is the most altered in cancer. Several decades of research have established that it is of pivotal importance in prompting neoplastic phenomena, including cancer initiation and progression. However, it has crucial functions for cellular life. Knowledge and awareness about these multifaceted properties should be part of the cultural background of all scientists. In this review, we describe and discuss the multifaceted roles of p53, from its discovery to clinical applications in cancer therapy.

**Abstract:**

p53 tumour suppressor gene is our major barrier against neoplastic transformation. It is involved in many cellular functions, including cell cycle arrest, senescence, DNA repair, apoptosis, autophagy, cell metabolism, ferroptosis, immune system regulation, generation of reactive oxygen species, mitochondrial function, global regulation of gene expression, miRNAs, etc. Its crucial importance is denounced by the high percentage of amino acid sequence identity between very different species (Homo sapiens, Drosophila melanogaster, Rattus norvegicus, Danio rerio, Canis lupus familiaris, Gekko japonicus). Many of its activities allowed life on Earth (e.g., repair from radiation-induced DNA damage) and directly contribute to its tumour suppressor function. In this review, we provide paramount information on p53, from its discovery, which is an interesting paradigm of science evolution, to potential clinical applications in anti-cancer treatment. The description of the fundamental biology of p53 is enriched by specific information on the structure and function of the protein as well by tumour/host evolutionistic perspectives of its role.

## 1. Introduction

In this scoping review, we describe the main mechanisms by which p53 regulates cell proliferation to understand how p53-oriented therapies can work as anticancer treatments. p53 discovery is reported as one of the most interesting paradigms of scientific debate and evolution (Section 2, p53 discovery). Then, p53 structure and function are described (Section 3, p53: structure and function), since they are related to important cellular processes (Section 4, p53: cellular processes and post-translational modifications; 4.1 Cell cycle arrest, 4.2 Senescence, 4.3 DNA repair, 4.4 Apoptosis), mainly regulating proliferation. In Section 5 (p53 mutations leading to tp53), we aim at dissecting the mutational landscape of p53, which is related to innovative therapeutic approaches against cancer. In Section 4 and Section 5, an intriguing and original evolutionary approach (from the host and from the cancer perspectives) is presented to underline the importance of p53 biologic functions and its profound involvement in cancer. In Section 6 (p53 is not only a simple guardian of the genome: interrelations with miRNAs, lncRNAs, cancer cell metabolism, mitochondria and immune response), we describe some p53 functions that, even if indirectly involved in cancer progression, could be targets of anti-cancer treatments in the near future. In Section 7, an updated survey on p53-oriented drugs in cancer treatment is reported, highlighting the fundamental importance of understanding cancer biology to build new anti-cancer treatments. Finally, in Section 8 and Section 9, we report our conclusions and perspectives for future research. 

## 2. p53 Discovery

In the late 1970s, antibodies recognized the same protein with a molecular weight of 53,000 in extracts of (i) chemically induced sarcomas and leukemias, (ii) spontaneously transformed fibroblasts in BALB/c (Bagg ALBino genotype-c) mice, (iii) and cells transformed by SV40 (e.g., Simian Virus 40: SV40 large T antigen). In particular, SV40, like other viruses bearing an oncogenic potential (Adenovirus type 2: adenovirus E1B; Human Papilloma Viruses types 16 and 18: HPV 16 and 18 E1B; etc.), stimulates the proliferation of infected cells in order to increase viral replication and virions assembly. This component was not detected in normal adult mouse fibroblasts, lymphoid and hematopoietic cells, normal mouse embryos, or 3T3 cells; it was designated as p53 [1]. This protein did not share antigenic determinants with the “large T” antigen of SV40, and it was later isolated from embryonic carcinoma cells in mice not infected by SV40. Thus, it was supposed to derive from a cellular and not from a viral gene [2]. 

Later, monoclonal antibodies against p53 were used to study the intracellular location of the protein by immunofluorescence assays. The experiments demonstrated p53 in all nuclei of transformed mouse cell lines; once again, no p53 was detected in normal mouse fibroblasts, 3T3 cells, bone marrow, thymus or embryo cells [3]. The expression of p53 in human cells was related to the growth characteristics of the cellular culture, being high levels of p53 associated with rapid cell proliferation and low p53 levels, with cessation or reduced cell division. For this reason, for the first decade following its discovery, the tp53 (tumour) protein was considered to be encoded by a proto-oncogene, stimulating cell growth and survival when forcibly expressed in cell lines [4]. 

It is now clear that the initial research describing tp53 function was inadvertently performed on mutant tp53 genes rather than the wild-type form. The awareness that p53 was actually not an oncogene but rather the “opposite”, namely a tumour suppressor, emerged many years later. 

The observation that SV40 drives tumorigenesis through the overproduction of p53 in cells seemed to lead, very logically, to the conclusion that p53 was a positive inducer of neoplastic transformation. In fact, the results of Linzer DI et al. demonstrated that the SV40 A gene product was required to initiate and possibly maintain the high levels of p53 protein found in virus-infected and virus-transformed cells. Furthermore, the cellular levels of p53 were high when T-antigen was functional but lower when T-antigen was non-functional, implying that p53 was correlated to neoplastic transformation [5]. Many other studies seemed to corroborate this role of p53 [6,7]. Starting from these assumptions, many researchers tried to confirm that p53 had oncogenic properties. The cloning of p53 from tumours or virus-transformed cell lines continued to prompt these conjectures at the beginning of the 1980s [8]. Using DNA clones and starting from the assumption that p53 overexpression contributed to the tumorigenic processes, the consequences of such overexpression were next assessed in several experimental models. These findings posed the basis of a series of studies, carried out in the early 1980s, which revealed that transfected p53 could cooperate efficiently with a number of established oncogenes (e.g., RAS) to transform primary cells in culture. In fact, it was demonstrated that p53 cooperated with the activated RAS oncogene to transform normal embryonic cells. The resulting neoplastic foci contained cells with markedly altered morphology and high levels of p53; these cells were efficiently able to cause tumours in syngeneic animals [9,10]. In sum, by the mid-1980s, p53 was generally acknowledged as an oncogene with a still unclear mechanism of action. 

However, several studies provided increasing proof that p53 was a tumour suppressor rather than an oncogene. In fact, in some models, including the Abelson murine leukemia-transformed cell line, it was noted that the p53 gene was heavily altered/rearranged by retroviral insertions more likely to disrupt protein functions [11,12,13]. It took several years to definitively accept this “contrasting theory”. This perspective became increasingly strong. In 1991, Halevy and coworkers demonstrated that in chemically induced tumours and cell lines, the overproduction of p53 was related to the mutant nature of the protein, leading to the overproduction of stabilized/not functioning p53. In fact, the presence of “wild-type” p53-inhibited cancer [14]. Mutant p53 genes were directly tested in a number of cases, by laborious and pioneering PCR-amplified cDNA cloning. Other studies gave similar results; it emerged that while mouse tumour-derived p53 mutants promoted cell transformation, wtp53 (p53) clearly did not. Only p53 cDNAs carrying such mutations (tp53) exerted transforming activities in experimental settings [15]. In the late 1980s, the evidence demonstrated that p53 loss from a frequent deletion involving the 17p chromosome in colon and lung cancers was responsible for neoplastic transformation along with a missense mutation in the remaining allele [16,17]. These data definitively suggested that p53 was a tumour suppressor gene. Forced expression of the wtp53 was able to block oncogene-mediated transformation in many different experimental models. Furthermore, spontaneous cancers occurred in 100% of p53 knockout (p53−/−) mice that also displayed strong susceptibility to γ-irradiation and carcinogen-induced tumours [18]. Studies confirmed that DNA clones of the wild-type p53 inhibited potent oncogenes (E1A and RAS) able to transform primary rat embryo fibroblasts [19,20]. Altogether, these data finally indicated p53 as a bona fide tumour suppressor gene.

## 3. p53: Structure and Function

Knowledge about p53 structure and conformation is important because, very recently, drugs aiming to restore p53 correct/wild-type folding have been designed. The p53 tumour suppressor is a flexible molecule composed of four identical protein chains. Flexible molecules are difficult to study with X-ray crystallography because they do not form ordered crystals and, even if they crystallize, the experimental images are often indistinct. Therefore, p53 was studied in fragmented parts, removing the flexible regions and determining the structure of the portions that form stable structures. These compact globular portions, called “domains”, have been well studied over time. The human p53 protein contains 393 amino acids and has been divided structurally and functionally into five domains with specific hot spots for mutations in human cancer: (1) an acidic amino-terminal domain (aa ~1–61), which is required for transcriptional activation, (2) a proline-rich domain (aa ~64–92), (3) a central core sequence-specific DNA-binding domain (aa ~100–300), (4) a tetramerization domain (aa ~323–355) and (5) a C-terminal regulatory domain (aa ~364–393) [21]. The transactivation domain (TAD) is located at the N-terminus and is subdivided into two regions: TAD1 and TAD2 (aa residues ~1–40 and ~40–61, respectively). These domains allow the binding of p53 to different cofactors and both are required for p53-mediated suppression of tumorigenesis in response to stress, such as acute DNA damage, oncogene activation, hypoxia and replication/translation stress activate sensor proteins, including ATM, ATR, Chk1, Chk2, DNA-PK and p14ARF. Nonetheless, each of the two transactivation domains confers to p53 the cofactor binding specificity that, in turn, influences the cell response to a specific type of stress. Examples of genes under p53 control are reported in Table 1.

It has been demonstrated that in the context of the acute DNA damage response and RAS oncogene expression, disruption of TAD1 abolishes the p53 response. On the other hand, TAD2 disruption retains similar wild-type functions capable of inducing cell cycle arrest and apoptosis. The simultaneous deletion of TAD1 and TAD2 completely abolishes p53 function, resulting in a p53 null response [22]. These findings indicate that specificity for gene transcription is provided by TADs. The TAD region allows the binding of its main negative regulator, MDM2 protein, which is encoded by the mouse double minute 2 (MDM2) gene. The importance of negative regulation of p53 by MDM2 is highlighted by the evidence that the homozygous deletion of MDM2 in mice results in complete embryonic lethality [23]. MDM2 is an E3 ubiquitin ligase targeting p53 at the C-terminal domain for proteolysis by the proteasome. Furthermore, human MDM2 and adenovirus E1B-55 kDa proteins also negatively regulate the p53 transcriptional activity through binding to the N-terminus activation domain [24,25]. The second domain of the p53 protein contains a proline-rich domain (PRD), also known as the polyproline (PP). Although some studies show that the PRD is required to suppress colony formation of tumour cells in vitro [26], many others show that it does not [27,28,29]. However, this region is of great importance for p53 stability as deletions/mutations in the PRD cause p53 nuclear export, becoming prone to ubiquitination and MDM2-mediated degradation [30]. Many studies suggest that PRD is essential for the efficient transcription function of p53 [31,32] and for the activation of that DNA damage-induced apoptosis and cell cycle arrest [33]. 

The third domain is the central core of p53 and it contains the DNA-binding domain (DBD). This region is protease-resistant and it contains a zinc ion, which is required for DNA binding activity. DBD is structured in a β sheet antiparallel, which in turn is structured in two α-helix interacting with DNA and allowing p53 to exert its function as a transcription factor. Specific target sequences are recognized, p53 REs [34]. These specific DNA sequences consist of two copies separated by 0–13 bp of 5′-RRRCWWGYYY-3′, where R is a purine, C is cytosine, W can be adenine or thymine, G is guanine and Y is a pyrimidine [35]. 

The tetramerization domain (TD) allows four p53 proteins to oligomerize as a tetramer conferring the appropriate protein conformation that binds to DNA [36]. Tetramerization of p53 is crucial for its full transcriptional function. Crystallographic studies suggest that a p53 monomer, which consists of a β strand and an α-helix, associates with a second monomer across an antiparallel β sheet and an antiparallel helix–helix interface to form a dimer. In turn, two of these dimers associate to form the tetramer [37,38]. Interestingly, the nuclear export signal (NES) of p53 is located within the tetramerization domain and is “shielded” in p53 tetramers, thus preventing their nuclear export [39]. The binding of p53 to DNA is highly cooperative both at the level of dimeric p53 and tetrameric p53; furthermore, oligomerization deficient mutants of p53 bind DNA with much lower affinities than the wild type [40,41]. The oligomerization is essential for the tumour-suppressive activity of p53. It was demonstrated that deletions in this region affect the ability of p53 to bind to DNA and influence the interaction with other proteins [42,43]. Finally, a very interesting study has shown that TD is useful for the efficiency of post-translational modifications of p53, such as ubiquitination and phosphorylation. Finally, it must be emphasized that MDM2 requires p53 in its oligomerized state to activate ubiquitination and degradation [44]. 

The C-terminal domain, rich in basic amino acids, regulates the ability of p53 to bind to specific DNA sequences. This domain can also bind non-specifically to different forms of DNA, such as DNA breaks or internal mismatches [45]. The C-terminal domain is a regulator domain subjected to post-translational modifications (required for p53 to change from an inactive conformation to an active conformation, allowing the DBD to bind to specific DNA sequences) [46]. In particular, these modifications include acetylation and phosphorylation. Without these modifications, partially induced by stress signals, this region inhibits the p53 DBD and, therefore, the protein does not work [47,48]. The C-terminal domain also contains the nuclear export and the nuclear localization signals. These two signals are important for p53 to exert its function as a transcription factor (in the nucleus) and to migrate to the cytoplasm (for its degradation). Finally, the C-terminal domain is the target of MDM2-mediated ubiquitination, which can occur in the nucleus [49,50] or in the cytoplasm [51]. A correct conformation of p53 is crucial for its function; mutations inducing misfolding are oncogenic and frequently found in several types of cancer.

## 4. p53: Cellular Processes and Post-Translational Modifications

Under normal conditions, in undamaged cells, the p53 protein is highly unstable (it has an average life of about 10–20 min), and it is present in very low concentrations. In fact, p53 protein continuously interacts with MDM2, which binds to the N-terminal end of the protein, as mentioned above. In general, the activation pathways of p53 are pleomorphic and involve both reduction in p53 degradation and several post-translational modifications (PTMs). Hundreds of p53 PTMs have been described. It is important to have a paramount view on this issue to understand two concepts: 1. PTMs are crucial in regulating the activity of p53, 2. the heterogeneity and reversibility of PTMs gives the opportunity to cells of making quick responses to varying environmental conditions. Several interesting reviews have already been published, and it is to these that we refer the readers [52,53,54]. Here, we will synthetically focus on ubiquitination, phosphorylation, acetylation, methylation, sumoylation and neddylation, which are the most studied and important PTMs. PTMs are generally reversible and can occur with or without genotoxic events. 

As already mentioned, the ubiquitin-proteasome pathway plays a major role in the regulation of p53, and, in particular, the MDM2-mediated poly-ubiquitination and nuclear degradation have a critical role in suppressing p53 function. Poly-ubiquitination occurs predominantly on Lys 48 and 29 and represents a so-called “molecular kiss of death”, prompting the proteasome-dependent degradation. Other proteins that ubiquitinate p53 are PIRH2 (p53-induced RING-H2), COP1 (Constitutively Photomorphogenic 1), synoviolin, Trim24 (tripartite motif-containing 24) and CARPs (caspase 8/10-associated RING proteins), all are E3-ubiquitin ligase that negatively regulates p53. P300 and CBP (Creb-Binding Protein) are considered as “multifunctional modulators” of p53 since they have both acetylase and poly-ubiquitin E4-ligase properties. However, ubiquitination has much more complex effects, since mono-ubiquitinations can be involved in regulating p53-mediated DNA repair, histone architecture, and cell cycle regulation. In fact, E4F1 (E4F transcription factor 1) is an atypical ubiquitin E3 ligase, and it activates an “oligo”-ubiquitination associated with cell cycle arrest. 

Interestingly, PTMs are associated to specific p53-related cellular effects; in particular, phosphorylation is the most studied modality of p53 activation. In fact, when the cell is subjected to genotoxic stress signals and various other types of DNA damages (e.g., γ and UV radiations, chemicals, heat, unexpected DNA replication errors, oxidative stress, etc.), protein kinases are activated, including ATM (Ataxia Telangiectasia Mutated), ATR (ATM and Rad3-related), PKC (protein kinase C), DYRK2 (Dual Specificity Tyrosine Phosphorylation Regulated Kinase 2), HIPK2 (homeodomain-interacting protein kinase), p38 and Cdk1 (Cyclin Dependent Kinase 1), which mediate p53 phosphorylation on serine residues, such as Ser15, Ser20 and Ser47. These modifications induce conformational changes and reduce the p53/MDM2 interaction, reducing the proteasomal degradation of p53 that accumulates. Furthermore, phosphorylation may trigger the interaction with proteins modulating specific cellular effects (i.e., proliferation and differentiation) involved in tumor progression. For example, kinases CK1d (Casein Kinase I delta) and CK11 (Casein Kinase 11) are able to phosphorylate Ser 6 and 9, inducing the interaction between p53 and SMAD (small mothers against decapentaplegic) proteins. Phosphorylation of NES in the TD is also involved in blocking the nuclear export of p53, determining its nuclear accumulation. 

Interestingly, acetylation and ubiquitination occur in the same Lys residues at the C terminus, and they are mutually exclusive. Acetylation of p53, via a reversible enzymatic process, protects p53 from degradation and allows its activation. Notably, Lys 120 and 164 acetylation sites are among the most common p53-mutated regions in malignant neoplasms; this observation indicates a crucial role of p53 acetylation in its tumor suppressive role. Methyl transferase enzymes (PRMT5—protein arginine methyltransferase 5, KMTs —lysine, K methyltransferases, SMYD2—SET And MYND domain containing 2, SET7/9, SET8—SET domain-containing proteins) can methylate several Lys and Arg residues of p53 during DNA damage response. The number and location of the methyl groups determine the final effect (activation or depression). The methylation is counterbalanced by the action of demethylases (particularly the Lysine Specific Demethylase 1 -LSD1-), contributing to regulate p53 activity. Sumoylation consists on the covalent ligation of SUMO (small ubiquitin-related modifier) groups at lysine residues. It occurs at Lys 386 by the TOPORS (TOP1 binding arginine/serine rich protein) and PIAS (protein inhibitor of activated stat) family members and it determines the export of p53 from the nucleus, prompting the accumulation of p53 into the cytoplasm. Similar to sumoylation, another process, neddylation, can be involved in conjugating ubiquitin-like proteins at Lys residues; it involves NEDD8 (neural precursor cell expressed developmentally downregulated protein 8). Neddylation does not modulate p53 degradation, rather it inhibits p53 activity. 

p53 plays an important role in many cellular processes, including cell cycle arrest, senescence, DNA repair, apoptosis, autophagy, cell metabolism, ferroptosis, generation of reactive oxygen species and global regulation of gene expression, each of them can potentially and heterogeneously contribute to its tumour suppressor function. 

### 4.1. Cell Cycle Arrest

**Figure 1:**
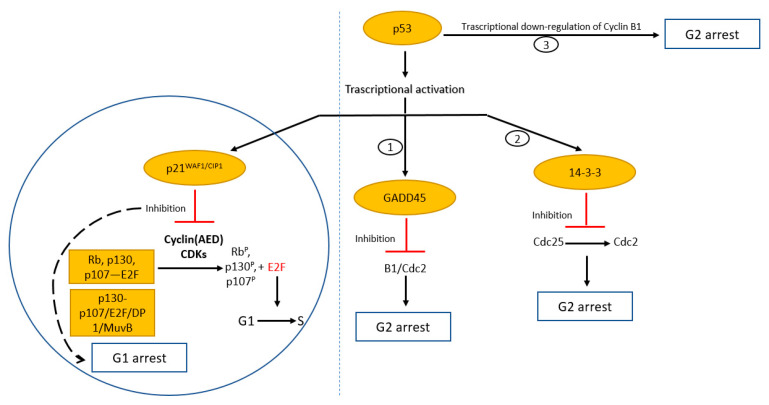
Cell cycle regulation by tumour suppressor p53. Evidence for both a G1 and G2/M checkpoint function. Transcriptional activation of p53 results in induction of p21WAF1/CIP1 that binds to and inhibits cyclin (AED)/cdk complexes. Hypo-phosphorylation of Rb and related proteins blocks E2F activity and concurs to the formation of the DREAM complex (p130/p107/E2F/DP1/MuvB), resulting in G1 arrest. Induction of GADD45 and 14-3-3 and repression of cyclin B1 cause cell cycle arrest in G2.

We believe that the control of cell proliferation and cellular senescence are two of the major properties of p53, accounting for its involvement in cancer. Furthermore, genes and related products involved in these p53-dependent pathways are often altered in cancer. The cell division process is highly ordered and regulated. The checkpoints are control points designed to delay the progression of the cycle in the next phase only when the previous stage is completed with the purpose of ensuring that daughter cells inherit a complete and faithful copy of the genome. p53 plays a key role in each of the cell cycle checkpoints with evidence for both a G1 and G2/M checkpoint function [55,56] (Figure 1).

The crucial target of p53 for arresting the cell cycle in the G1 phase is the factor p21WAF1/CIP1, which encodes a cyclin-dependent kinase inhibitor (CKI). In fact, a binding site for p53 is present in the promoter of the WAF1/Cip1 gene, showing that it is a p53-inducible gene [57]. p53 induces the transcription of p21, which, in turn, inhibits the cyclin complexes (A, E and D)/CDK (cyclin-dependent kinases), causing the inhibition of their kinase activity [58]. This does not allow the phosphorylation of the Rb (Retinoblastoma) protein, which remains bound to the transcription factor E2F and fails to activate E2F-responsive genes (G1 arrest). 

p53 can also indirectly repress the transcription of several genes involved in cell cycle progression. In fact, after p21 p53-dependent activation, hypophosphorylated pRB-related proteins (p107 and p130) facilitate DREAM (dimerization partner 1, retinoblastoma-like, E2F and MuvB) complex formation [59]. This represents the so-called “p53–DREAM pathway”. DREAM complex binds DNA predominantly through CHRs (cell cycle genes homology regions) and E2F sites, working as a potent transcriptional repressor complex. Interestingly, the number of repressed genes after p53 activation is greater than those promoted [60]. Some of the repressed genes are crucial to cell cycle arrest and apoptosis (e.g., cyclins A, B1, B2, CDK1, CDC20, BIRC5). 

p53 has also been implicated in the control of a G2/M checkpoint, and, in this case, there are three possible mechanisms. In the first, p53 induces the transcription of the Gadd45 gene, which inhibits the cyclin B/cdc2 kinase activity or disrupts the cyclin B1/Cdc2 complex (G2 arrest) [61,62]. In the second, p53 induces the transcription of 14-3-3, another protein involved in G2 arrest [63,64]. It binds and sequesters the phosphorylated Cdc25 in the cytoplasm, thereby preventing its activation [65]. Finally, the third mechanism appears to be a direct process of transcriptional down-regulation of cyclin B1 by p53 [66,67,68,69,70]. In fact, the cyclin B1/Cdc2 complex is the major regulatory factor activating the entry into mitosis [71,72].

The dysfunction of checkpoints leads to uncontrolled proliferation, accumulation of mutations, and, ultimately, progressive survival advantage. 

### 4.2. Senescence

**Figure 2:**
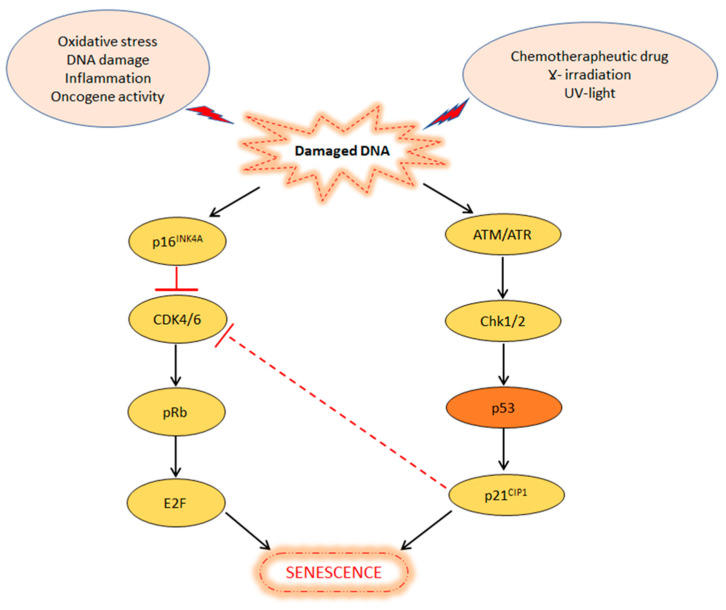
p53 and P16INK4A pathways in the induction of cellular senescence. Internal or external stress factors trigger the DNA damage response pathway. On one side, p16INK4A inactivates Cdk4/6, determining hypo-phosphorylation of Rb. On the other side, p53 induces p21, which blocks CDK4/6 or directly results in senescence.

One of the most important physiological barriers against cancer development is cellular senescence. It is defined as a stable and terminal state of growth arrest, triggered by stressful insults and certain physiological processes, in which cells are unable to proliferate despite normal growth conditions and mitogenic stimuli. Thus, cell senescence play an important role in tumour suppression, as well as in wound healing, and protection against tissue fibrosis [73]. Cells that are unable to undergo senescence and continue to proliferate, acquire chromosomal aberrations, which can lead to their malignant transformation. The main signals inducing senescence through the p53 pathway are DNA damage, oxidative stress, inappropriate activation of oncogenes, molecular chaperone depletion and chemotherapy [74,75]. Therefore, senescence can be intended as an adaptive response of cells and organisms to unfavorable internal or environmental conditions [76,77]. The main studied pathways involved in the regulation of cellular senescence are ATM/ATR (sensor proteins belonging to the superfamily of kinases) and p16INK4A/Rb tumour suppressor pathways [78] (Figure 2).

As already mentioned, various internal or external stresses trigger the DNA damage response pathway, which, in turn, activates the p16INK4A/Rb and/or ATM/ATR pathways. In the first case, p16INK4A protein inactivates Cdk4/6 causing hypo-phosphorylation of Rb and reduction in E2F activity, driving cell cycle arrest or senescence. In the second case, ATM/ATR sensor proteins activate Chk1/Chk2 kinases that transactivate p53 and p21CIP1, contributing to the G1 arrest or senescence. Moreover, p21CIP1 protein levels may lead to the inhibition of Cdk4/6 activity, of the first pathway, which contributes to the G1 arrest or senescence. Interestingly, G1 arrest or senescence depends on the severity and permanence of the damage [79]. In fact, if the damage is sustainable for repair, p53 causes cell cycle arrest and promotes the transcription of proteins involved in DNA repair (see next section); however, if the damage is extensive and cannot be repaired, p53 induces the transcription of proteins involved in senescence or other forms of cell death, such as apoptosis [80].

### 4.3. DNA Repair

**Figure 3:**
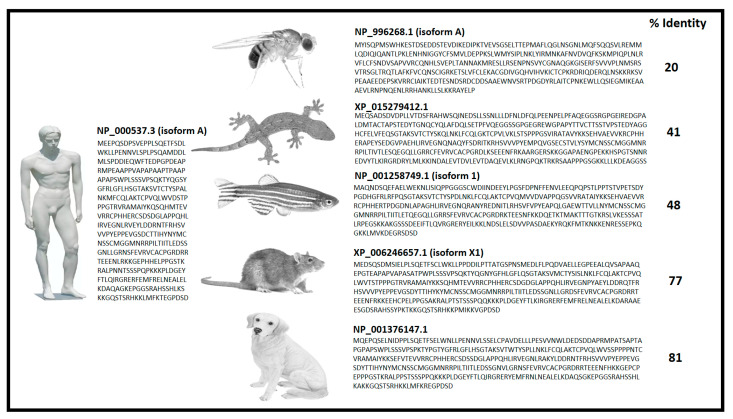
Percent of identity calculated with the Needleman–Wunsch sequences alignment tool (https://blast.ncbi.nlm.nih.gov/Blast.cgi accessed on 4 February 2022) of p53 from different species. Protein ID, isoform and amino acid sequences are depicted for each species.

One of the most profound connections between p53 and cancer development is represented by the involvement of p53 in supporting DNA repair. Knowledge about these connections is crucial to understanding the role of p53 in both cancer biology and therapy. In fact, cells can revert the large variety of DNA lesions that are induced by endogenous and exogenous genotoxic attacks using repair processes. From a descriptive and functional point of view, these DNA repair processes can be divided into nucleotide excision repair (NER), base excision repair (BER), mismatch repair (MMR), non-homologous end-joining (NHEJ) and homologous recombination (HR) [81,82]. Evolution of these mechanisms, which are able to preserve DNA integrity against radiation from the sun, from cosmic rays and from the earth’s crust, have been fundamental for the establishment of life. p53 is widely involved in the first three repair mechanisms (NER, BER and MMR). Its crucial importance is denounced by the high percentage of conformational similarity and amino acid sequence identity between very different species (Homo sapiens, Drosophila melanogaster, Rattus norvegicus, Danio rerio, Canis lupus familiaris, Gekko Japonicus) (Figure 3). However, in rodents, the sequence base composition of the p53REs (p53 responsive elements: DNA containing specific motifs with high affinity for p53 binding) is “highly” conserved for cell cycle, senescence and transcriptional regulation, compared to its primate counterparts (including humans). In contrast, the p53REs for most of the DNA repair and some apoptotic p53 target genes are poorly sequence conserved; therefore, those genes are less responsive to p53 transcriptional control. Interestingly, the degree of sequence conservation of p53REs among species accounts for different evolutionary contexts of p53 function and divergent biological priorities. 

NER is a DNA repair pathway, which can eliminate various helix-distorting DNA lesions, involving long strands of 2–30 nucleotides. These defects are generated mainly by environmental mutagens, such as ultraviolet light (UV) irradiation and chemical compounds [83]. One of the first pioneering studies was conducted by Smith ML et al., they showed that human cell lines with disrupted p53 function had significant losses of fitness and survival after UV irradiation [84]. In fact, a variety of helix-distorting lesions occurring after UV-induced DNA damage induce a rapid p53 activation, which, in turn, induces the expression of DDB2 and XPC proteins [85]. DDB2 associates with its partner DDB1 to form a heterodimer, which, in turn, binds to photoproducts (6-4PPs) and pyrimidine dimers (CPDs) and recruits the XPC protein during the early steps of NER [86]. The XPC protein recognizes and binds to DNA sites with disrupted/destabilized base pairs facilitating the binding of the multimeric protein complex TFIIH that plays a central role in NER via its helicase activity (ATPase/helicase subunits, XPB and XPD) [87].

BER is a multiphase damage repair process that involves non-bulky DNA lesions, mostly caused by reactive oxygen species. In this pathway, apurinic and apyrimidinic (AP)-endonucleases are key players allowing the removal of damaged bases and the subsequent repair of the sites. Several studies have shown the interaction in this repair process between the AP-endonucleases and p53 [88,89]. In particular, p53 is regulated by AP-endonuclease/redox effector factor 1 (APE1/Ref-1) via redox-dependent and redox-independent pathways. The function of APE1/Ref-1 is to promote the tetramerization and activation of p53 [90]. p53 can also positively regulate the expression of some additional BER genes at the transcriptional level, such as the gene for 8-oxoguanine glycosylase (OGG1) [91]. Wild-type p53 cell lines exposed to the same levels of reactive oxygen species (ROS) show more rapid removal of the 8-oxoguanine lesions from DNA, compared with p53-defective cells [92]. Noteworthily, p53 can also regulate the expression of MUTYH gene, which encodes an adenine DNA glycosylase involved in oxidative DNA damage repair of the BER pathway. This interaction is very interesting because, in recent years, MUTYH has been identified as responsible for some forms of hereditary cancers, including colorectal cancer [93,94,95]. 

DNA MMR is a highly conserved biological pathway that plays a key role in maintaining genomic stability. MMR and p53 functions are frequently lost in human tumours. A group of highly conserved MMR proteins whose transcription is genetically controlled by p53 mediates the repair of base/base mismatches and small insertion/deletion mispairs generated during DNA synthesis. MMR corrects genetic replication and recombination errors or insertion/deletion loops, which result in the formation of mismatched nucleotides following normal DNA replication or action of exogenous agents (e.g., anticancer therapies, such as cisplatin or alkylating agents). The MMR mechanism has been extensively characterized in E. Coli where MutS and MutL are involved in correcting mismatches of newly synthesized DNA [96,97]. In eukaryotes, MMR is initiated through the actions of two heterodimeric MutS homologs (MSH: MutS Homologue), MSH2-MSH3 and MSH2-MSH6 [98]. These two complexes recognize different types of mismatches; MSH2-MSH6 is able to recognize large deletions and insertions, while MSH2-MSH3 is able to recognize small deletions. The relationship between p53 and MMR seems to be centered on the MMR core component MSH2. In fact, the MSH2–MSH6 complex can, at least in vitro, enhance the binding of p53 to DNA substrates with topological distortions, and this activity depends on the phosphorylation state of p53 [99]. Furthermore, recently, it has been shown that p53 signaling is suppressed in MSH2-deficient cells [100].

Ultimately, if severely damaged DNA cannot be repaired, p53 strongly activates and induces cell elimination through apoptosis.

### 4.4. Apoptosis

**Figure 4:**
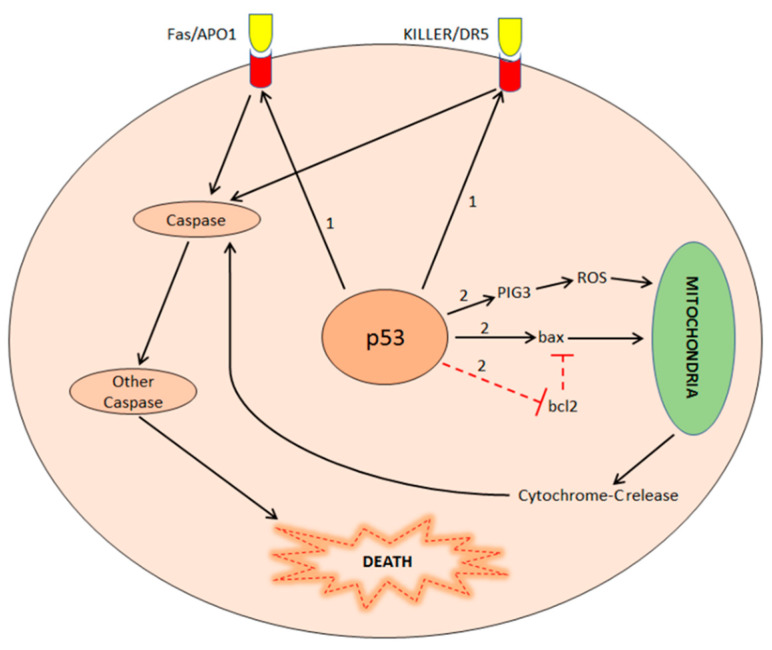
Apoptosis induction by p53 in extrinsic (1) and intrinsic (2) pathways. In the extrinsic pathway, p53 activates Fas/APO1 and KILLER/DR5 genes that result in the expression of two membrane receptors, activating the caspase cascade and cell death. In the intrinsic pathway, p53 induces pro-apoptotic proteins (PIG3, bax) and inhibits anti-apoptotic proteins (bcl2). Pro-apoptotic proteins induce cytochrome-C release by mitochondria and activation of the caspase cascade and cell death.

Besides the control of the cell cycle, p53 activation is one of the most important stimuli to induce apoptosis (regulated and programmed cell suicide). The final effect is dependent upon the type and extent of cellular damage and the cellular context. The loss of p53-dependent apoptosis strongly contributes to cancer development, particularly by promoting the accumulation of mutations and aberrant cell functions. Several genes controlled by p53 are involved in inducing apoptosis. Among these genes, the most important are Bax, members of the Bcl-2 family, Fas/APO1, KILLER/DR5 and PIG [101]. However, the mechanism by which p53 causes the apoptosis of cells affected by irreparable damages is complex and it includes multiple pathways (Figure 4).

In the extrinsic pathway, p53 induces the transcription of Fas/APO1 and KILLER/DR5, death receptors located on the cell membrane, which, in turn, activate the “caspase cascade” resulting in cell apoptosis [102]. In particular, the expression of KILLER/DR5 increases after exposure of wild-type p53-expressing cells to DNA damaging agents, such as γ-radiation or chemotherapy [103]. Instead, in the intrinsic pathway, p53 induces the transcription of pro-apoptotic proteins, such as Bax, and inhibits the transcription of anti-apoptotic proteins, such as Bcl2. Cellular effects are mitochondria damage and cytochrome-C release and, ultimately, apoptosis [104]. Furthermore, in the intrinsic pathway, p53 activates the transcription of the PIG3 protein, which induces oxidative stress (an increase in reactive oxygen species concentration), resulting in apoptosis [105].

## 5. p53 Mutations Leading to tp53

**Figure 5:**
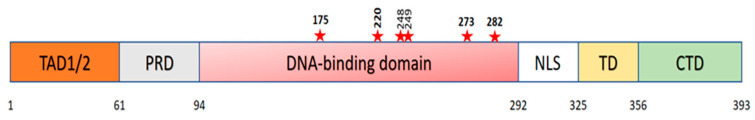
Structure of p53 protein showing the different domains and hot spots (red stars) mutations occurring in human cancer. The 393 amino-acid p53 protein is depicted from the amino-terminus (1) to the carboxy-terminus (393) with boundaries for various domains shaded with different colors: transactivation domain (TAD1/2), proline-rich domain (PRD), DNA-binding domain (DBD), nuclear localization signal (NLS), tetramerization domain (TD), C-terminal domain (CTD).

p53 is the most frequently mutated gene in human malignant neoplasms. Knowledge of the most representative mutations (including classes and some specific types) can improve both diagnostic and therapeutic appraisal of oncologists about several types of cancer. Cancer-derived p53 mutants include missense (~74%), truncation, frameshift and deletion mutations. The most common somatic alterations are represented by point mutations (more complex rearrangements are less frequent) being missense mutations predominant. Among these missense mutations, approximately 80% occur in the p53 DBD (exons 5–8), and several “hotspot mutations” have been identified in this domain, such as Arg-175, Tyr-220, Gly-245, Arg-248, Arg-249, Arg-273, and Arg-282 [106] (Figure 5).

Within this region, the most frequent mutations, are divided into two categories: (1) “conformational mutations”, such as Arg-175-His (p.R175H), Tyr-220-Cys (p.Y220C), Gly-245-Ser (p.G245S) and Arg-249-Ser (p.R249S), which lead to structural changes in the binding domain, and (2) “contact mutations”, such as Arg-248-Gln (R248Q), Arg-273-His (R273H) and Arg-282-Trp (R282W), which alter the ability of the protein to bind DNA [107]. The most frequent mutations in all human cancers are p.R248Q, p.R273H and p.R175H. The vast majority of p53 mutants lose the wild-type function or exert a ‘dominant-negative’ effect on the wild-type allele products; these mutations impair the activation of p53 target genes involved in suppressing tumour growth (loss of function, LOF). 

The loss of DNA integrity guardian function of p53 is a critical event that prompts cancer mutational plasticity. In fact, cancer is a multi-gene disease prompted by DNA mutations, which are also the fuel of genetic variability and humans’ evolution [108]. Therefore, tumours arise as a consequence of a physiologic phenomenon (mutations). In humans, the mutational rate (new mutations in a gene during the time encompassing two generations) is very low (estimated to be approximately 10^−4^ to 10^−6^) [109]. In fact, “beneficial” mutations (defined as a stable gain of fitness-associated function) occur and stabilize through thousands of generations [110]. The reason for it being such a long time is that eukaryotic cells, over millions of years, have acquired quick and effective DNA damage repair machinery to survive the cosmic radiation and natural mutagens. Most malignant cancers are necessarily characterized by the alteration of genes related to the DNA repair (i.e., p53) to increase their mutational plasticity and phenotypic heterogeneity (angiogenic switch, epithelial to mesenchymal transition, immune system evasion, migration, invasion, etc.) much faster than expected. In fact, genetic analysis through modern and high-throughput genome sequencing techniques (next generation sequencing—NGS) shows a mutation rate of malignant cells ever exceeding that of normal cells [111]. This is an adaptive mechanism of tumour cells in order to resist to the microenvironmental defensive mechanisms that strongly limit their proliferation and metastatization. From an evolutionary point of view, the acceleration of the mutational rate is finalized to the development of new cell functions during the development of the tumour. Some of these mutations are deleterious and can induce cell death but others are favorable and protect cells from external injuries (i.e: immune system). Much higher is the mutational rate and much more probable is the possibility that favorable mutations arise in a cancer mass. Therefore, the loss of p53 provides cancer with a great advantage in terms of evolution towards growth and adaptation to the changing environment.

Interestingly, some p53 mutations, such as p.R248W and p.R249S, have been surprisingly associated to a ‘gain-of-function’ (GOF) promoting cancer malignancy (invasion and metastasis), genetic heterogeneity and chemoresistance [112,113]. These mutants are able to form ternary complexes with some transcription factors (e.g., NF-Y and p300) that promote DNA synthesis, cell cycle progression and cancer cell proliferation [114,115,116,117]. Very recently, it has been demonstrated a unique mechanism for the GOF activity of p53-specific hepatocellular carcinoma (HCC) mutant p.R249S through c-Myc activation [118]. Furthermore, some p53 mutants are specifically involved in prompting cancer metabolism and suppressing autophagic cell death [119,120,121,122]. These data emphasize the wide range of GOFs involving tp53. We refer the readers to other interesting reviews for the insight into the impact of different p53 mutations on cancer [123,124].

In conclusion, from a functional point of view, mutations commonly affecting tp53 can be divided into two types: LOF and GOF mutations. They are a paradigm of biological and genetic complexity deserving a differential therapeutic approach in the perspective of innovative anti-cancer strategies. 

## 6. p53 Is Not Only a Simple Guardian of the Genome: Interrelations with miRNAs, lncRNAs, Cancer Cell Metabolism, Mitochondria and Immune Response

### 6.1. p53, miRNAs and lncRNAs

**Figure 6:**
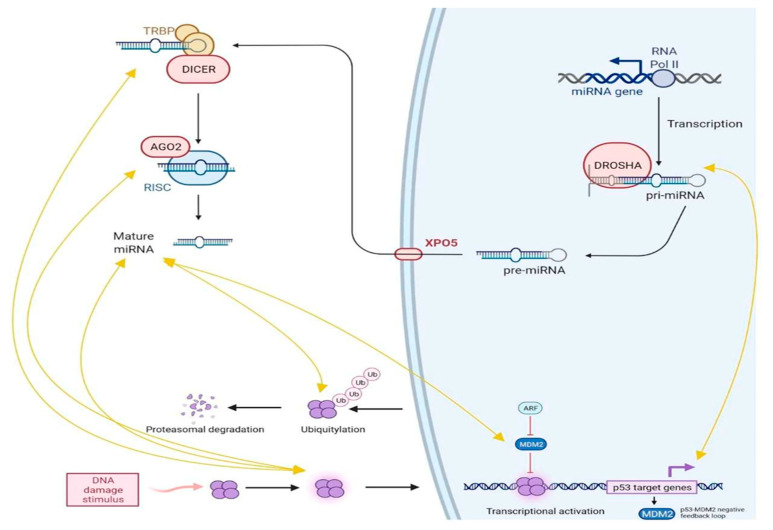
Crosstalk between p53 and miRNAs (see text for details).

Micro RNAs (miRNAs) and long non-coding RNAs (lncRNAs) belong to the non-coding RNA family (piwi-interacting RNAs, enhancer RNAs, circular RNAs, etc.), meaning that those transcripts are not directly coding for proteins. We believe it is important to deepen insight into the miRNAs and lncRNAs biology and their relationships with p53, since they are an exciting and innovative field of potential anti-cancer drugs. In fact, it is increasingly clear that p53/miRNAs/lncRNAs pathways are involved in regulating cancer phenotype (proliferation, migration, invasiveness, etc.). Most importantly, both miRNAs and lncRNAs “mimics” and “repressors” can be built to overexpress or silence the transcripts function, respectively. Briefly, miRNAs biogenesis is divided into two phases: transcription and maturation (Figure 6). The primary miRNA (pri-miRNA) sequence is encoded in either protein-coding or noncoding human genome region and is transcribed normally during the DNA replication as a sequence of 800–1000 nucleotides still attached to the chromosome. Afterward, the pri-miRNA is processed by the Drosha RNase enzyme, which is able to create a 70 nucleotide-long looped structure from the whole transcript; this sequence is called pre-miRNA and is obtained after the first step of the maturation process. Pre-miRNA are actively transported from nucleus to cytoplasm in order to continue the maturation through a RanGTP/Exportin-3 complex; then, another RNase, Dicer, cleaves the pri-miRNA and generates the mature miRNA, normally showing 20–24 bp in length [125,126]. Mature miRNAs are subsequently included in the miRNA-induced silencing complex (miRISC) together with the Argonaute RNA binding proteins in order to exert their modulation activity thanks to the binding with messenger RNAs (mRNAs), transcribed by protein-coding genes. In particular, miRNAs can bind preferentially to the complementary mRNA 3′-UTR region, in order to stop their translational capability [127]. 

It is important to keep in mind that one miRNA can bind to different mRNAs and vice versa, making those interactions difficult to predict in their final outcome. This is why miRNAs expression patterns have gained more and more importance during the last decades, since they are often double-tied with specific cell phenotypes and peculiarity, possibly exploitable even in cancer therapy [128]. A direct consequence of the above description is the double possible miRNAs behavior within the cancerous cell, as oncomiRs or tumour suppressors [129]. 

Regulation exerted by miRNAs could either suppress p53 protein levels by targeting the relative mRNA 3′-UTR region or increase its expression by repressing its negative regulators. A wide range of microRNA families is capable of directly regulating p53, most of them by 3′-UTR binding and the prevention of relative mRNA translation. Some remarkable examples are miR504, miR125b and miR30d, which were identified as p53-related oncomiRs, capable of directly downregulating p53 expression and correlated to an aggressive cancer behavior in both human and animal models [130,131]. In recent years, some of those cancer-related miRNAs have been extensively studied, and some of them showed other possible indirect modulation of the p53 levels and pathway activation. MiR25, for example, exerts direct and indirect regulation of the p53 pathway by interacting with other important players, such as MDM2, MYC and E2F1, or by its capability to transcriptionally regulate the p53 gene-enhancing cell proliferation and invasiveness [132]. 

On the other hand, p53 itself can modulate miRNAs expression (Figure 6), serving as a trans-activator of oncosuppressive miRNAs and downregulating oncogenic miRNAs in order to suppress cancer progression. This is the case of miR34a and miR29, whose expression is directly correlated to p53 activation, and the effects aim to stop aberrant cell growth and restore cell cycle integrity and physiological conditions [133,134]. Recent research has also revealed that mutant p53, highly represented in cancerous lesions, controls the production of certain miRNAs in order to obtain carcinogenic activities. For example, mutant p53 upregulates the production of oncogenic miR-128-2 in breast cancer cells by binding to the promoter of its host gene, in order to enhance cell proliferation and survival [135]. Furthermore, mutant p53 binds directly to the promoters of numerous tumour-suppressive miR-NAs, inhibiting their production in cancer cells. The direct consequence is both wild-type and mutant p53 capability to interfere with miRNAs maturation and biogenesis [136]. In detail, interacting with RNase Drosha, p53 is able to potentiate the pri-miRNA processing, resulting in a higher tumour suppressor miRNA expression, while mutant p53 possibly acts in the opposite direction both inhibiting maturation and blocking p72/p68 regulatory activities and p63-mediated Dicer expression [137]. As briefly summarized, p53 and miRNAs are double tied and their peculiar interplay is crucial for homeostasis maintenance and cancerogenesis as well. The interaction loops between those masters intracellular regulators, together with other non-coding RNAs, above all long non-coding RNAs, are today widely studied worldwide, since RNA-based therapy is a fact, and all the evidence in vivo is incredibly boosting this topic into its translation in human healthcare.

**Figure 7:**
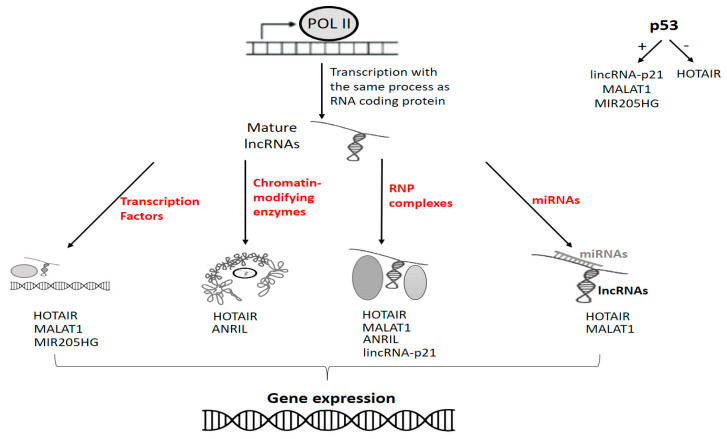
In red are indicated the main interactions accounting for lncRNAs gene expression regulation. Some oncogenic lncRNAs (upper right side of the figure) are positively or negatively regulated by p53.

More than 30000 lncRNAs have been described in humans [138]. Their biogenesis is similar to that of mRNA (canonical pathway): synthesis by RNA polymerase II (Pol II) and maturation through capping, polyadenylation, and splicing. However, they can be synthetized through several non-canonical processes [139]. Different genomic origins have been described: sense, antisense, bidirectional, intronic and intergenic. Ribosomal RNA (rRNA) and transfer RNA (tRNA) involved in the translation process formally belong to the long non-coding RNA family. However, lncRNAs commonly refer to noncoding transcripts longer than 200 nucleotides involved in the regulation of gene expression. Mechanisms of action have been elsewhere detailed [140], and they are based on interactions with DNA, RNA and proteins (Figure 7).

Several lncRNAs involved in tumour progression are transcriptionally activated or depressed by p53. The most studied examples are: lincRNA-p21, MALAT1 (metastasis-associated lung adenocarcinoma transcript-1), HOTAIR (HOX transcript antisense RNA) and MIR205HG (MIR205 host gene) [141,142,143,144,145]. Interestingly, both p53-related miRNAs and lncRNAs can be involved in driving neoplastic phenomena. An example is the involvement of lincRNA-p21 and miR-155 in coordinating the neoplastic adaptive dynamics of HIF-1α (hypoxia-inducible factor-1) in response to hypoxia [146]. Furthermore, the relationships between lncRNA can be complex and indirect. For example, ANRIL (antisense noncoding RNA in the INK4 locus), an oncogenic lncRNA, is involved in the repression of the p14ARF. CDKN2A/p14ARF is crucial in inducing cell cycle arrest in G2 and apoptosis being the most important physiological inhibitor of MDM2. Thus, the silencing of CDKN2A activity through ANRIL has biological effects similar to loss of p53 [147,148]. It is argued that mut-p53/lncRNAs/miRNAs pathways can disrupt cellular homeostasis and drive tumour development. However, the relationships between mutant forms of p53 and non-coding RNAs are extremely complex and still largely unknown.

### 6.2. p53 and Cancer Metabolism

In addition to the aforementioned well-known functions, many of which are inherent in cell survival control, p53 performs numerous and not less important effects on cellular metabolism [149]. A complete dissertation of connections between p53 and cancer metabolism is beyond the scope of this review. 

However, it is important to know that p53 is capable of promoting oxidative phosphorylation and inhibiting anaerobic glycolysis, acting on genes, such as SCO2 (synthesis of cytochrome oxidase 2) and GLS2 (mitochondrial glutaminase), which promote mitochondrial oxidative phosphorylation, and TIGAR (tp53 inducible glycolysis and apoptosis), which inhibits glycolysis [150,151]. Moreover, p53 downregulates both glycolysis and glucose transport within the cell through the inhibition of the GLUT4 receptor. In addition, p53 stimulates the β-oxidation of fatty acids (FAs) in mitochondria and blocks the FAs biosynthesis acting on FASN (fatty acids synthase) and ACLY (ATP citrate lyase). At the same time, p53 negatively regulates the biosynthesis of nucleotides and proteins, and it reduces the activity of the enzyme glucose-6-phosphate dehydrogenase (G6PD), which has an important role in initiating the pentose phosphate pathway (PPP). The above-mentioned functions favor the resting of the cells and the quiescence phase [152]. 

**Figure 8:**
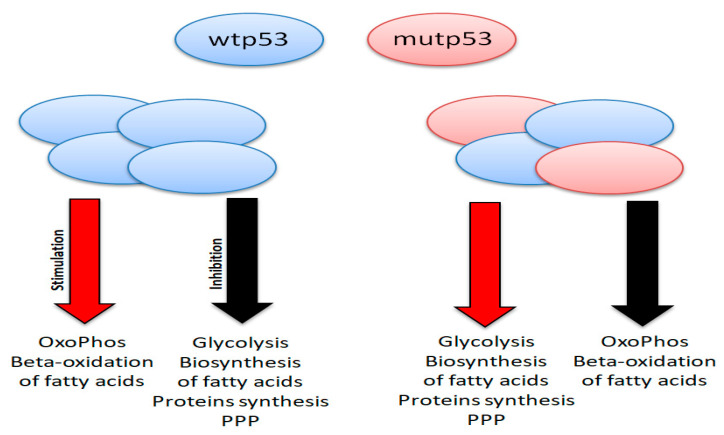
Opposite functions on cancer metabolism of wt (wild-type) p53 and mut (mutant) p53.

Interestingly, in some GOF mutations (e.g., p.R175H, p.P151S), tp53 promotes metabolic functions in a manner that is “opposite” to the wild-type protein (Figure 8). The result is a strong increase in anaerobic glycolysis (to the detriment of oxidative phosphorylation), a typical phenomenon of neoplastic cells called the “Warburg effect”, namely the production of ATP through the anaerobic glycolysis also in presence of oxygen. In addition to the Warburg effect, mutant p53 is also capable of promoting the PPP, which results in an increase in the biosynthesis of nucleotides and fatty acids, and importantly it is able to increase the synthesis of proteins in the cells. The increased synthesis of FAs, which leads to the increase in the membrane phospholipids, proteins and nucleotides, ultimately supports cell proliferation, providing cells with “raw materials” to be able to duplicate themselves continuously. The increase in anaerobic glycolysis provides energy in the form of ATP more rapidly than can occur with oxidative phosphorylation. The process also becomes independent from the oxygen support (in fact, the vessels generally do not grow with the same speed of tumour masses), thus responding to the continuous demand for energy by cancer cells [153]. Thus, many GOF mutations of the tp53 gene, very frequent and “early” in malignant cells, in addition to dysregulating the cell cycle in favor of greater proliferation, modify the metabolism of the neoplastic cells making it capable of adapting to their excessive proliferation. 

Understanding the cross-talk between p53 and cellular metabolism may lead in the next future to the integration of p53-oriented therapies with the modulation of metabolic pathways for the development of more effective therapeutic strategies.

### 6.3. p53 in Mitochondria

In order to increase the readers’ awareness about the extremely pleomorphic roles of p53, we believe it to be useful to briefly describe the involvement of p53 in mitochondria dynamics. Mitochondria are intracellular organelles where the oxidative phosphorylation occurs. It consists on the oxidation of glucose and subsequent production of Adenosine 5′-triphosphate (ATP, the most important form of chemical energy usable by eukaryotic cells). Mitochondria contain DNA (mtDNA) as a 16.6-kb, circular, double-stranded molecule encoding 37 genes (13 respiratory enzyme complex polypeptides, 22 transfer RNAs and 2 ribosomal RNAs) [154]. As already explained, most cancer cells need a high rate of glucose uptake because they depend on the anaerobic metabolism of glucose (with production of lactic acid in the cytoplasm), even with sufficient oxygen levels (“Warburg effect”). However, specific cancer clones may use the oxidative phosphorylation as main energetic source (so-called “metabolic reprogramming”). It has been demonstrated that this phenomenon may depend on onco and tumour suppressor gene mutations and it confers to malignant cells a favorable metabolic plasticity. Actually, mitochondrial deregulation is considered as one of the most important cancer hallmarks. Notably, the morphology, the number and the activity of mitochondria are tightly controlled by the nucleus through crosstalk signaling pathways. Interestingly, the transcriptional landscape modified by p53 proteins has a strong impact on several mitochondrial characteristics [155]. In response to classical p53-inducing stimuli, in addition to transcriptional activation into the nucleus of BAK and BAX proteins (which activate MOMP—mitochondrial outer membrane permeabilization—factors), a fraction of the p53 protein localizes to mitochondria. Here, p53 is able to physically interact with (i) anti-apoptotic members of the Bcl2 family, and (ii) procaspase-3 stimulating the MOMP process (with subsequent release of Cytochrome C in the cytoplasm and trigger of apoptosis), and (iii) cyclophilin D (cypD), the key regulator of the mitochondrial PTP (permeability transition pore), inducing necrosis mainly upon oxidative stress [156]. These are all interesting examples of “post-translational” mechanisms of p53 action. Alterations of these mechanisms can contribute to malignant transformation preserving malignant cell clones from apoptosis/necrosis and prompting progression. Recently, mitochondrial dynamics in terms of fission (mitochondrial division) and fusion have been implicated in cancer metastatic phenotypes [157,158]. Fission is regulated by mTORC1 f (mammalian target of rapamycin aomplex 1), which increases the fraction of free eIF4E (by phosphorylating 4E-BPs), which, in turn, translates MTFP1 (mitochondrial fission process 1), a transmembrane mitochondrial protein [159]. MTFP1 activates, through phosphorylation, DRP1 (dynamin-related protein 1) that mediates outer mitochondrial membrane fission. p53 prevents oncogenic activation of the mTORC1/MTFP1/DRP1 pathway while p53 loss determines mitochondrial fragmentation and increased activity of proliferative signaling pathways [158]. Other oncogenic mechanisms involving mitochondria are out of the scope of this review, acting predominantly in p53-independent manner (e.g., ROS and/or succinate overproduction) [160]. 

Beside the regulation of apoptosis/necrosis, p53 is involved in controlling the mitochondrial volume–density (biogenesis) and integrity. These characteristics are related to the cellular needs of ATP hydrolysis during oxidative stress and are regarded as adaptation phenomena mediated by the expression of p53R2 (p53-controlled ribonucleotide reductase). Recently, a p53-inducible protein called MIEAP (mitochondria-eating protein) has been described as a crucial factor driving the repair or degradation of damaged mitochondria (mitochondrial quality control function) [161]. Finally, it is increasingly evident that p53 contribute to the mitochondrial genome integrity through its translocation into mitochondria and interactions with mtDNA repair proteins [162].

### 6.4. p53 and Immune Response

The era of anti-cancer immuno-therapies has just started, and several drugs have been already approved for anti-cancer treatment (e.g., immune-checkpoint inhibitors). In this new and expanding context, oncologists should be aware that a cross-talk exists between p53 and the immune system. The relationships between cancer and the immune system are composite and complex and concur to dynamically shape cancer phenotypes in all phases of malignant transformation. Interestingly, both innate (NK cells and macrophages) and adaptive (T cells) immune responses are involved in the early recognition and elimination of transformed cells (“immune surveillance”) [163,164]. However, other cell types are paradoxically involved in promoting tumour progression (Tregs, MDSCs, macrophages and neutrophil subsets) [165]. Oncogenic events are able to directly or indirectly modulate the multiple aspects of the immune response through several mechanisms, including their antigenicity, influence on antigen presentation machinery, cytokines production, lymphocytes migration, receptor expression, etc. 

p53 is involved in a cross-talk with the immune system by influencing both humoral and cellular components of the immune response. p53 is involved in regulating the activity of chemokine (CCL2, CXCL17) [166,167], chemokines receptors (CXCR4 and 5) [168,169] and cytokines (IL-6) [170] by negatively affecting STAT3 (signal transducer and activator of transcription 3) and related signaling pathways [171]. Thus, the loss of p53 function in cancer can (i) increase the activity of cytokines and their receptors (migration and proliferation), (ii) sustain an abnormal response to inflammatory stimuli (angiogenesis and matrix degradation), and (iii) facilitate a tumour-promoting microenvironment [172,173,174]. Furthermore, in highly and persistently inflamed sites (where cancer initiation is favored), interferons (IFNs) are able to stimulate p53, which, in turn, determines apoptosis and cell division arrest. On the other hand, it has been demonstrated that free radicals produced by neutrophils infiltrating inflamed sites (as in inflammatory bowel diseases) are prone to induce mutations in p53 [175]. Some mutant p53 proteins inducing GOFs (e.g., p53 R273H) bind to NF-κB subunit p65 determining hyper-activation of IL-1β signaling [176] that, in turn, associates with increased tumour proliferation and angiogenesis, and immune-suppressive microenvironment.

The transcriptional activity of p53 is fundamental in modulating the cellular immune system. In fact, recent advances revealed that p53 controls the expression of proteins involved in immune responses, including ERAP1, TAP1, ULBP1 and 2, miR-34a, and TLR3 and 9. Interestingly, p53-mediated transcription in cells exposed to different stressing stimuli determines an increase in the expression of MHC-I/peptide complexes. By contrast, p53 mutations associate with reduced antigen presentation. These effects are due, in part, to the transcriptional control of ERAP1 and TAP1. ERAP1 is an aminopeptidase associated with the endoplasmic reticulum (ER) involved in processing antigen precursors, subsequently transported by TAP1 (transporter associated with antigen processing 1) into the ER (where they bound to MHC class I) [177]. ULBP1 and 2 are ligands of NKG2D, a C-type lectin-like receptor, mainly expressed on CD8+ T, NK and γδ T cells. It is involved, in concert with other costimulatory molecules, in the full activation of effector cells, even if its mechanism is still incompletely understood. Contradictory results have been obtained on the interplay between p53 and ULBP1 and 2, since it has been demonstrated that p53 can either induce [178] or reduce [179] the expression of these two NKG2D ligands. 

T cells activity in physiologic conditions is strictly regulated, particularly to avoid auto-immunity (self-tolerance). This equilibrium is maintained through a complex and redundant system based on stimulatory and inhibitory signals. The inhibitory signals belong to the so-called “immune checkpoints”. One of the most studied immune checkpoints is represented by programmed death-ligand 1/programmed death-1 (PD-L1/PD-1) [180]. PD-1 is predominantly expressed on immune cells, including T, B and NK cells and subsets of dendritic cells (DCs). PD-L1 is widely expressed in non-cancerous tissues. The binding of PD-L1 to PD-1 delivers into immune effectors a potent inhibitory signal reducing or even preventing their activation. In the last decades, the overexpression of PD-L1 in cancers has been described as one of the most important mechanisms of tumour immune escape. Interestingly, p53 can suppress PD-L1 expression through miR-34a (its direct transcriptional target). Consequently, perturbation of p53 (mutations or loss) increases PD-L1 expression, which, in turn, suppresses T cell activities [181].

Toll-like receptors (TLRs) are both surface and intracellular proteins able to bind several pathogen-derived substances, including nucleic acids from viruses, proteins and lipids from bacterial and fungal species. TLR3 and 9 (two intracellular TLRs) are transcriptional targets of p53 [182,183]. The induction of TLRs expression concurs to induce apoptosis in infected cellular environments. Most cancer cells bearing mutant p53 are unable to achieve this type of response against infections [184].

Beside the role in acting as a regulatory factor of the immune response, p53 can itself elicit an immune response. Interestingly, it has been demonstrated that the presence of T cell clones against normal/wild-type p53 does not induce auto-immunity and reduces the growth of mutant p53 tumours [185,186]. Thus, T cells armed against wild-type p53 epitopes recognize both mutant and wild-type p53 tumours. However, T cell clones recognizing specific neo-epitopes from mutant p53 (not present in the wild-type form) have also been isolated from epithelial cancer patients [187], suggesting that an immune response against TP53 is built. Taken together, these data represent the premise to develop anti-cancer p53-oriented immunotherapies (see Section 7). 

## 7. p53-Oriented Therapies in Cancer Treatment

p53 can be exploited for anti-cancer treatment. In fact, in the last fifteen years, anti-cancer research has been oriented on attempting (i) to restore wtp53 activity, (ii) to eliminate tp53 over-expressing cancer cells, or (iii) to influence tp53 conformation (particularly when a GOF is involved). Exploring active clinical trials, “recruiting” or “completed” in the clinicaltrials.gov database provides a fascinating and paramount view of the clinical efforts toward p53-oriented therapies. Selected trials can be displayed as examples of that (Table 2). 

Replacement of defective p53 through adenovirus-mediated gene therapy (recombinant adenoviral human p53—rAd-p53) is the most applied treatment in very different solid tumours (breast, colon, lung cancers, soft tissue sarcomas, melanomas, lymphomas, etc.). RAd-p53 has been used in monotherapy (NCT02429726, NCT00004038, NCT00004041, NCT00003588, NCT00003167), in association with radio-chemotherapy (NCT02429037), with radiotherapy (NCT00004225), with chemotherapy (NCT00894153, NCT00902122, NCT00902083, NCT02435186), with surgery (NCT01574729) or biologic therapies (NCT03544723, NCT02842125, NCT00776295). Unfortunately, rAd-p53 needs to be administered locally through intra-peritoneal, intra-pleural, and/or intra-lesion injections to avoid infections out of the tumour targets and optimize efficacy. Most of these trials are phase I studies with MTD (maximum-tolerated dose) and DLT (dose-limiting toxicity) as primary end-points and the assessment of tumour shrinkage as secondary or exploratory ones. No definitive results have been so far published. In this context, the example of a non-viral carrier of normal/effective p53 is SGT-53, a complex of cationic liposome encapsulating a human wtp53 DNA sequence in a plasmid backbone (NCT02340117: a phase II study of SGT-53 plus gemcitabine and nab-paclitaxel in advanced pancreatic cancer; NCT02340156: a phase II study of SGT-53 in recurrent glioblastoma). 

The elimination of p53-positive cancer cells is pursued in many trials whose eligibility criteria for enrolment is the evidence of p53 immuno-histochemical tumour over-expression. Therapeutic strategies include the use of tp53-derived peptides with cytokines or immunologic adjuvants (NCT01639885, NCT00001827, NCT00844506), adoptive transfer of ex vivo reactivated tp53 specific T cells (NCT02577588), modified vaccinia virus Ankara expressing tp53 (tp53-MVA) with (NCT03113487, NCT02432963) or without immune-checkpoint inhibitors (NCT01191684) or chemotherapy (NCT02275039), infusion of autologous dendritic cells pulsed ex vivo with mutated tp53-derived peptides (NCT00019916, NCT00978913, NCT00019929, NCT00617409), vaccination with adenovirus-based tp53 vaccine (NCT00082641, NCT01042535), infusion of ex vivo tp53 T-cell receptor transduced peripheral blood lymphocytes (NCT00393029), infusion of ALT-801 (a bi-functional fusion protein comprising interleukin-2 linked to a soluble, single-chain T-cell receptor domain that recognizes a peptide epitope aa264–272 of the tp53 antigen displayed on cancer cells in the context of HLA-A*0201) (NCT00496860, NCT01029873). Most of these studies are phase I/II trials. Definitive data have been published about NCT01191684 and NCT00496860 studies. In the NCT01029873 phase I trial [188], six colon and five advanced and chemotherapy-refractory pancreatic cancer patients were p53-MVA immunized. There were no adverse events > grade 3 according to NCI common toxicity criteria. CD4+ and CD8+ T cells recognizing a p53 peptide repertoire consistent with vaccination were detectable after the first immunization. However, the frequency of PD1+ T cells in patients’ peripheral blood was inversely correlated with the peak of CD8+ p53 response. Interestingly, anti-PD1 in vitro increased the immune responses against tp53, suggesting that the association of tp53MVA with immune checkpoint inhibitors could be a future successful strategy to enhance immunity against p53 mutant cancers. In the NCT00496860 phase I/II trial [189], ALT-801 was given to p53+/HLA-A*0201 patients with metastatic cancers (four daily 15-min i.v. infusions followed by ten days rest and four additional administrations). Patients were treated at different doses: 0.015, 0.040 and 0.080 mg/kg/dose. Two DLTs were registered (grade 4 thrombocytopenia and myocardial infarction) in the 0.08 mg/kg patients cohort, thus the MTD was 0.04 mg/kg. Patients treated at the MTD experienced toxicities similar to those associated with high-dose IL-2 but of lesser severity. Interestingly, the treatment-induced IFN-γ (interferon-γ) but not TNF-α (tumour necrosis factor-α) increase. Twenty-six patients with different solid tumours were treated (eleven melanoma, nine renal cancer, two prostate, one colon, one neuroendocrine tumour, one head and neck, and one non-Hodgkin lymphoma). Ten patients experienced stable disease while a patient with metastatic melanoma displayed a complete absence of metastatic disease after resection of responsive lesions. Therefore, ALT-801 is safe and displays potential anti-tumour properties. 

The third strategy relies on influencing p53 regulation by re-activating its functions through (1) inhibition of p53 negative control or (2) “conformational cure”. The most applied strategy in the first case is represented by MDM2 and HDM2 (p53-specific E3 ubiquitin ligase) inhibition. Some small orally available molecules have been produced to selectively block p53/MDM2/HDM2 interaction and inhibit p53 ubiquitination and subsequent degradation in many different solid tumours. Mechanistically, these molecules increase p53 concentrations, strongly activating p53-mediated apoptosis in cancer cells retaining wild-type p53. Representative trials are NCT01760525 (CGM097, HDM2 inhibitor), NCT05180695, NCT02143635 (HDM201, HDM2 inhibitor), NCT03781986, NCT03611868 (APG-115, MDM2 inhibitor), NCT03975387 (ASTX295, MDM2 inhibitor), NCT03217266 (navtemadlin, MDM2 inhibitor) and NCT02264613 (ALRN-6924, MDM2 inhibitor). The studies are ongoing and definitive data have been recently published only for CGM097 (NCT01760525). There were no DLTs and the MTD was not determined in 51 metastatic patients refractory to standard therapies receiving oral treatment with CGM097 10–400 mg 3qw or 300–700 mg 3qw 2 weeks on/1 week off. Delayed-onset thrombocytopenia was the most frequently observed toxic event. An interesting disease control rate was observed (39%), with one partial response in a patient with malignant melanoma and nineteen patients with stable disease [190].

Some molecules are able to reactivate p53 by restoring the normal structure (“p53 reactivators”). Examples of this therapeutic approach are PC14586, p28 and APR-246. The first molecule binds to the crevice of mutant p.Y220C restoring its conformation (NCT04585750) and normal function [191]. The azurin-derived cell-penetrating peptide p28 (a redox protein isolated from Pseudomonas aeruginosa) binds to the DBD of p53 restoring anti-proliferative and pro-apoptotic activity (NCT0 1975116) [192]. The APR-246 compound is spontaneously converted into the active form (methylene quinuclidinone) and is able to covalently bind to cysteine residues in mutant p53, inducing stabilization of the protein toward a normal conformation (NCT02098343, NCT03268382).

## 8. Conclusions

p53 has highly pleiotropic roles, mainly regulating the transcription of genes involved in cell growth control. The protein surprises us every year as new pathways are discovered increasing the importance it plays in the life of cells. Interestingly, but not surprisingly, p53 is the most altered gene in cancer. Somatic (missense) mutations (tp53) are found in more than 50% of human malignancies and even when not directly involved, in the remaining 50%, there is a high probability to find alterations in p53 regulators and/or effectors. It represents often an “early” molecular gain for prompting cancer genetic plasticity and evolution towards adaptation to the surrounding environment (cancer “beneficial” mutations). The last issue makes p53-oriented therapies difficult to apply and, unfortunately, in most cases, late. Therapeutic strategies aimed to target p53 in cancer treatment can be considered in the “early days” of their development. However, it is now clear that p53 inactivation and/or abnormal GOFs are essential for the development of nearly all cancers. 

## 9. Perspectives

Integrated diagnostics based on extensive and high profile molecular technologies for identifying the early phases of p53-mutated cancers are needed. On the other hand, multi-targeted associated therapies, including p53-oriented interventions, immuno-therapies and tumour signal inhibitors, to treat the advanced phases, deserve in the next future intensive clinical exploration, taking the advantage of next generation sequencing, allowing rapid, reliable and comprehensive genetic characterization of tumours.

## Figures and Tables

**Table 1:** Examples of transcriptionally activated target genes of p53 and their function.

Cell Cycle Regulation and Apoptosis		DNA Repair and Stress	Cell Growth and Angiogenesis	Transcription Regulation	Signal Transduction	Biosynthesis and Metabolism
AEN	PLK2/3	APOBEC3C	CSF1	ATF3	CERS5	CES2
BAX	PMAIP1	ASCC3	FOSL1	GPR87	FAM198B	CPE
CCNG1	PPM1D	BTG2	GDF15	PRDM1	FAM13C	FUCA1
CDIP1	SAC3D1	DDB2	KITLG	WDR63	FAM210B	GLS2
CDKN1A	SPATA18	ENC1	IER5	ZNF79	LAPTM5	ISCU
CYFIP2	SULF2	FBXO22	PADI4	ZNF219	PHLDA3	NADSYN1
DRAM1	TNFRSF10B	FBXW7	PGF	ZNF337	PLCL2	NTPCR
DUSP14	TP53INP1	MDM2	SERPINB5	ZNF561	RRAD	PANK1
EDA2R	TRIAP1	MICALL1	TGFA		TLR3	PGPEP1
EPHA2		POLH			TSPAN11	PRKAB1
FAS		RRM2B			ZMAT3	RPS27L
FDXR		SESN1/2				SCO2
GADD45A		TM7SF3				TIGAR
GRHL3		TMEM68				
IKBIP		TRAF4				
LIF		XPC				

Genes were partially derived from Fischer M. (Census and evaluation of p53 target genes. https://doi.org/10.1038/onc.2016.502 accessed on 14 March 2022). p53 target genes were selected from both individual studies and throughput datasets (repressed or “contradictory results” genes were not included) and only genes activated in at least 6 out of 16 genome-wide data sets were reported. Visit www.targetgenereg.org (accessed on 14 March 2022) tool to explore relationships between specific genes and p53.

**Table 2:** Examples of clinical studies exploring p53-oriented therapies in solid tumors.

Study ID	Phase	Drug	Mechanism of Action
NCT02429726	II	Recombinant adenoviral human p53	Replacement of defective p53.
NCT00004038	I		
NCT00004041	I		
NCT00003588	I		
NCT00003167	I		
NCT02429037	II		
NCT00004225	I		
NCT00894153	IV		
NCT00902122	IV		
NCT00902083	IV		
NCT02435186	II		
NCT01574729	II		
NCT03544723	II		
NCT02842125	I/II		
NCT00776295	II		
NCT02340117	II	SGT-53 (cationic liposome encapsulating p53)	
NCT02340156	II		
NCT01639885	II	Vaccine from tp53-derived peptides	Immune-mediated elimination of p53 mutated neoplastic clones.
NCT00001827	II		
NCT00844506	II		
NCT02577588	I	Adoptive transfer of ex vivo reactivated p53 specific T cells	
NCT03113487	II	Modified vaccinia virus Ankara vaccine expressing p53	
NCT02432963	I		
NCT01191684	I		
NCT02275039	I		
NCT00019916	I/II	Autologous peripheral blood-derived antigen-presenting cells pulsed in vitro with p53-derived	
NCT00978913	I		
NCT00019929	II		
NCT00617409	II		
NCT00082641	I/II	Autologous dendritic cells pulsed with adenovirus p53	
NCT01042535	I/II		
NCT00393029	II	TP53/T-cell receptor transduced peripheral blood lymphocytes	
NCT00496860	I/II	ALT-801	Induction of immune response against p53+ cells. The drug is a bifunctional fusion protein comprising interleukin-2 linked to a soluble, single-chain T-cell receptor domain that recognizes a peptide epitope (aa264–272) of the human p53 antigen displayed on cancer cells in the context of HLA-A*0201 (p53+/HLA-A*0201).
NCT01029873	I/II		
NCT01760525	I	CGM097	MDM2 inhibition.
NCT05180695	I/II	HDM201	
NCT02143635	I/II		
NCT03781986	I/II	APG-115	
NCT03611868	I/II		
NCT03975387	I/II	ASTX295	
NCT03217266	I	Navtemadlin	
NCT02264613	I/II	ALRN-6924	
NCT04585750	I/II	PC14586	Small molecule “reactivating” p53. It binds to the crevice of mutant p.Y220C p53, restoring the normal structure and tumor suppressing function.
NCT01975116	I	Azurin-derived cell-penetrating peptide p28	After preferentially penetrating cancer cells, azurin induces a post-translational increase in p53 by inhibiting its ubiquitination.
NCT02098343	I/II	APR-246	It binds to cysteine residues in mutant p53, thereby producing thermo dynamic stabilization of the protein and shifting equilibrium toward a functional conformation.
NCT03268382	I		

Only therapeutic studies were selected. Diagnostic, prognostic or early detection studies were not included. Clinical trials whose status was “unknown” or “withdrawn” or “suspended” were not included into this table (reporting only active or completed trials).

## Data Availability

Not applicable.
